# A review on the biological roles of LncRNA PTCSC3 in cancerous and non-cancerous disorders

**DOI:** 10.1186/s12935-023-03037-y

**Published:** 2023-08-29

**Authors:** Majid Ghasemian, Jafar Poodineh

**Affiliations:** 1 Department of Clinical Biochemistry, Faculty of Medicine, Ahvaz Jundishapur University of Medical Sciences, Ahvaz, Iran; 2 Pediatric Gastroenterology and Hepatology Research Center, Zabol University of Medical Sciences, Zabol, Iran

**Keywords:** PTCSC3, Cancer, Thyroid, Signaling pathway, Tumor suppressor

## Abstract

Long non-coding RNA papillary thyroid carcinoma susceptibility candidate 3 (LncRNA PTCSC3) is located on human chromosome 14q13.3. PTCSC3 functions as a tumor suppressor lncRNA to regulate essential cellular processes such as apoptosis, cell proliferation, migration, invasion, angiogenesis, and epithelial-to-mesenchymal transition. PTCSC3 is also involved in the regulation of the Wnt/β-catenin signaling pathway, aerobic glycolysis, and p53 pathways. Downregulation of PTCSC3 has been associated with an increased risk of many tumors such as thyroid, gastric, laryngeal, breast, cervical, oral, lung, and glioma cancers. In addition, dysregulation of PTCSC3 has been reported in non-cancerous disorders notably osteoporosis and periodontitis. However, a number of single nucleotide polymorphisms at PTCSC3 have been linked to a higher risk of human diseases. This literature review summarizes the diagnostic, prognostic, and the clinical value of abnormal expression of PTCSC3 in cancerous and non-cancerous disorders and comprehensively analyzes potential molecular regulatory mechanism related to PTCSC3, which is expected to provide clear guidance for future PTCSC3 research.

## Introduction

Recent advances in whole-genome sequencing technology demonstrate that the majority of human genes encode non-protein-coding RNAs (ncRNAs) rather than proteins [1, 2]. The two main types of regulatory ncRNAs include microRNAs (miRNAs, < 30 nts) and long non-coding RNAs (lncRNA, > 200 nts) [3]. The role of miRNAs is to regulate gene expression by binding to the 3’- untranslated region (3’- UTR of the target messenger RNAs (mRNAs), resulting in translational inhibition or mRNA cleavage [4]. LncRNAs are transcribed by RNA polymerase II (RNAPII), and have the ability to regulate gene expression at different levels, including transcriptional, post-transcriptional, and epigenetic, using a variety of processes, including chromatin remodeling, genomic imprinting, binding to transcription factors, and mRNA or protein interactions [5, 6]. Previous studies have shown that pathological conditions alter the lncRNAs’ normal function, and their aberrant expression can interfere with normal biological functions by causing abnormal cell proliferation, apoptosis, migration, invasion, drug resistance, and cell cycle disruption [7, 8].

LncRNA papillary thyroid carcinoma susceptibility candidate 3 (PTCSC3) is located on human chromosome 14q13.3 and has four exons and a transcript with 1152 nucleotides in length (https://ensembl.org). PTCSC3 was first identified to be downregulated in thyroid cancer, where it acts as a tumor suppressor lncRNA [9]. Additionally, PTCSC3 is located near the SNP rs944289 (3.2 kb downstream of rs944289), which this SNP suppresses PTCSC3 expression by destroying a transcription factor-binding site in the promoter and increases the susceptibility to thyroid cancer [9, 10]. Despite many reports on PTCSC3’s role in the pathogenesis of both cancers and non-cancerous diseases, there is currently no comprehensive and detailed overview of PTCSC3. This literature review, for the first time, comprehensively summarizes the research progress of PTCSC3, molecular regulatory mechanisms, and clinical significance. This will help to clarify PTCSC3’s crucial role and offer guidance for PTCSC3 studies in the future.

## The expression of PTCSC3 in different cancers

The tumor-suppressive function of PTCSC3 has been documented in several cancers including thyroid, gastric, laryngeal, breast, cervical, oral, lung, and glioma cancers.

### Thyroid cancer (TC)

**Fig. 1:**
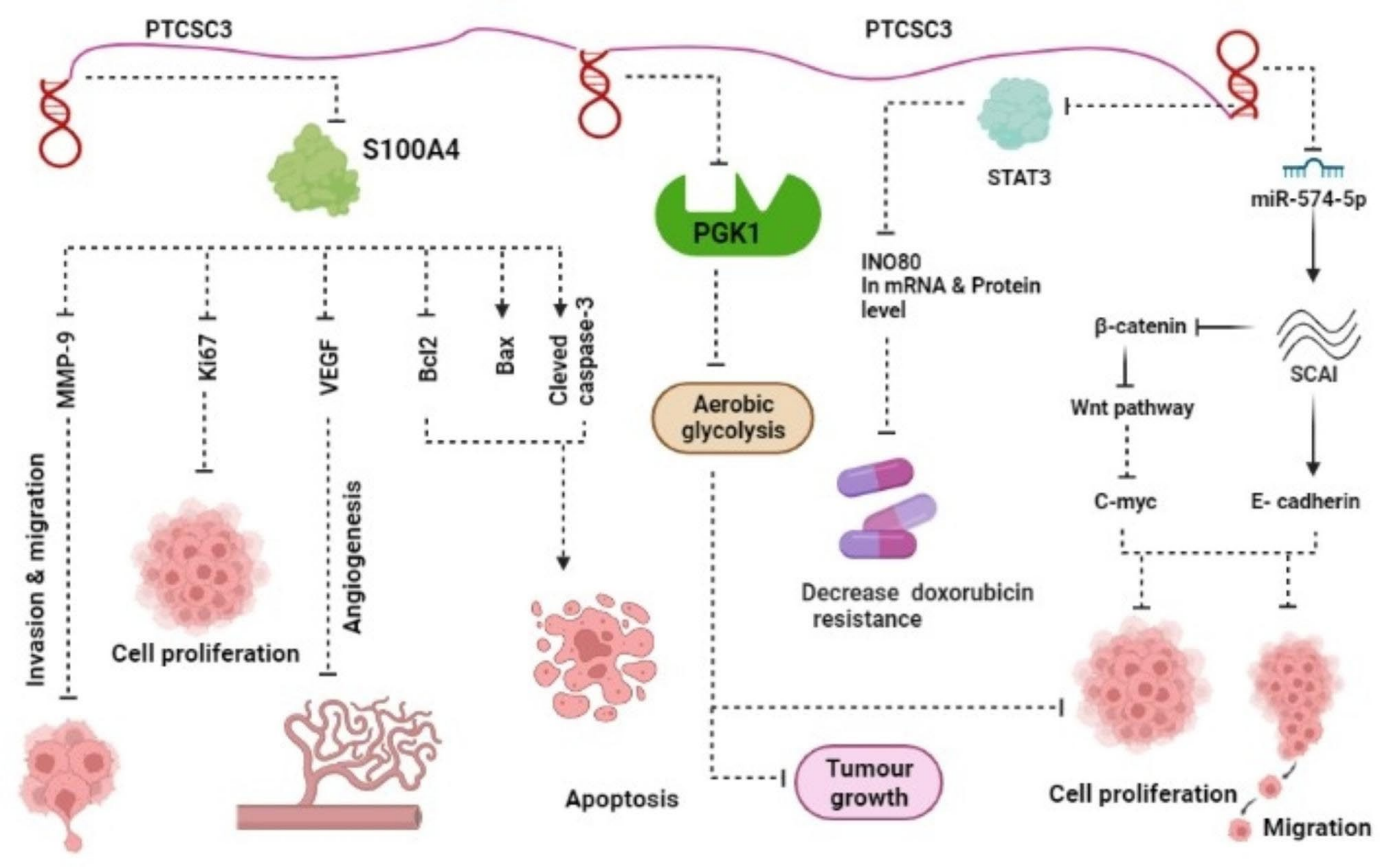
The tumor suppressive role of PTCSC3 in thyroid cancer. PTCSC3 can effect on cell proliferation, angiogenesis, invasion, migration, apoptosis and drug resistance through regulating downstream genes

PTCSC3 was first identified to be downregulated in TC tissues and malignant cell lines, and this finding was confirmed in several subsequent studies [11–14]. The function of lncRNA PTCSC3 in TC is schematically summarized in Fig. 1. It has been suggested that the suppressor of cancer cell invasion (SCAI) has a tumor-suppressive function in TC because it inhibits the myocardin-related transcription factor (MRTF), which regulates cancer cell invasion. In addition, SCAI can regulate the activity of the Wnt signaling pathway. In the study conducted by Wang et al., it was shown that the expression levels of SCAI and PTCSC3 were both suppressed, while β-catenin and miR-574-5p were promoted in papillary thyroid cancer (PTC) tissues and cell lines. Because PTCSC3 sponges miR-574-5p and SCAI is a miR-574-5p target gene, overexpressing PTCSC3 could lower miR-574-5p expression levels, upregulate SCAI, and inhibit both in vitro and in vivo cell proliferation and migration [15–17]. In line with these results, Fan et al. reported that PTCSC3 can act as an endogenous decoy for miR-574-5p in TC cells, where following overexpression of PTCSC3, cell proliferation was decreased while apoptosis and cell cycle arrest were improved [11]. Another function of PTCSC3 in TC cells is to reduce drug resistance; PTCSC3 overexpression could decrease doxorubicin resistance in anaplastic thyroid cancer (ATC) cells by downregulating the oncogenic transcription factor, signal transducer and activator of transcription3 (STAT3). In addition, inhibition of STAT3 could reduce the expression of INO80 at both mRNA and protein levels. INO80 is an ATP-dependent chromatin remodeling complex, which is involved in drug resistance [13]. Therefore, PTCSC3 inhibits INO80 expression by negatively regulating STAT3, resulting in decreased drug resistance. Jiang et al. reported that overexpression of PTCSC3 remarkably suppressed the Warburg effect (aerobic glycolysis) in PTC cells by directly interacting with phosphoglycerate kinase 1 (PGK1). As a result of this interaction, the progression of tumor cells was inhibited both in vitro and in vivo [18]. Moreover, PTC cells have been shown to express high levels of S100A4, a calcium-binding protein involved in cancer progression. Overexpression of PTCSC3 could result in the downregulation of S100A4 and its target genes, such as MMP-9 and VEGF, leading to increased apoptosis and decreased cell proliferation, invasion, migration, and angiogenesis [19]. In accordance with the previous study, Jendrzejewski et al. reported that as a result of ectopic expression of PTCSC3, the expression levels of S100A4, VEGF, and MMP-9 were all decreased. They came to the conclusion that the PTCSC3 reduces cell motility and invasion by decreasing S100A4 expression [12] (Table 1).

**Table 1:** The role of PTCSC3 in different cancers

Cancer type	Clinical samples	Assessed cell line	PTCSC3 expression	Effect in vitro	Regulatory mechanism	Ref
TC	10 paired tissues	PTC − 1, Nthy-ori3-1	Down	Proliferation ↓, Migration ↓ Tumor growth ↓	PTCSC3/miR-5745p/SCAI/Wnt/β catenin	(17)
TC	73 paired tissues	BCPAP, TPC-1	Down	Invasion ↓, Motility ↓	PTCSC3/ S100A4/VEGF/ MMP-9	(12)
TC	-	BCPAP, FTC133, 8505 C	Down	Growth ↓, Cell cycle ↓, Apoptosis ↑	PTCSC3/miR-574-5p.	(11)
TC	20 paired tissues	8505 C, FTC 238, FTC 133	Down	Drug resistance ↓	PTCSC3/ STAT3/ INO80	(13)
TC	68 paired tissues	PTC − 1, Nthy-ori3 -1, BCPAP	Down	Aerobic glycolysis↓, Proliferation ↓ Tumor growth ↓	PTCSC3/ PGK1	(18)
TC	-	SW579, TPC-1, BCPAP, K1, Nthy-ori3 -1	Down	Proliferation ↓, Invasion ↓ Migration ↓, Angiogenesis ↓	PTCSC3/ S100A4	(19)
GC	78 paired tissues	SNU-1, Hs 746T	Down	Proliferation ↓ Stemness↓	PTCSC3/ lncRNA Linc-pint	(25)
GC	68 cases and 60 controls	SNU-1, AGS	Down	Proliferation ↓, Invasion ↓ Migration ↓	-	(23)
GC	80 paired tissues	AGS, SNU-1	Down	Migration ↓, Invasion ↓	PTCSC3/ lncRNA HOXA11-AS	(22)
GC	77 paired tissues	SNU-1, AGS	Down	Invasion ↓, Migration ↓	PTCSC3/ HULC/ Wnt	(20)
CC	40 paired tissues	Caski, Siha, Hela, C4-1	Down	Proliferation ↓, Invasion ↓	PTCSC3/ miR-574-5p	(37)
CC	30 paired tissues	Hela, C-33 A	Down	Proliferation ↓, Invasion ↓ Migration ↓	PTCSC3/ miR-574-5p	(36)
TNBC	69 paired tissues	BT-549, HCC70	Down	Proliferation ↓	PTCSC3/LncRNA H19	(34)
OC	15 paired tissues	SCC-4, SCC-9 SCC-15, SCC-25	Down	Proliferation ↓, Invasion ↓ Apoptosis ↑	PTCSC3/ Bax, Bcl-2 PTCSC3/ LC3B-I, Beclin 1	(38)
LSCC	66 cases, 52 controls	UM-SCC-17 A	Down	Proliferation ↓	PTCSC3/ LncRNA HOTAIR PTCSC3/STAT3	(31)
Glioma	-	U251,U87, SHG44, SHG139	Down	Proliferation ↓, Invasion ↓ Migration ↓, EMT ↓	PTCSC3/LRP6/Wnt	(40)

### Gastric cancer (GC)

**Fig. 2:**
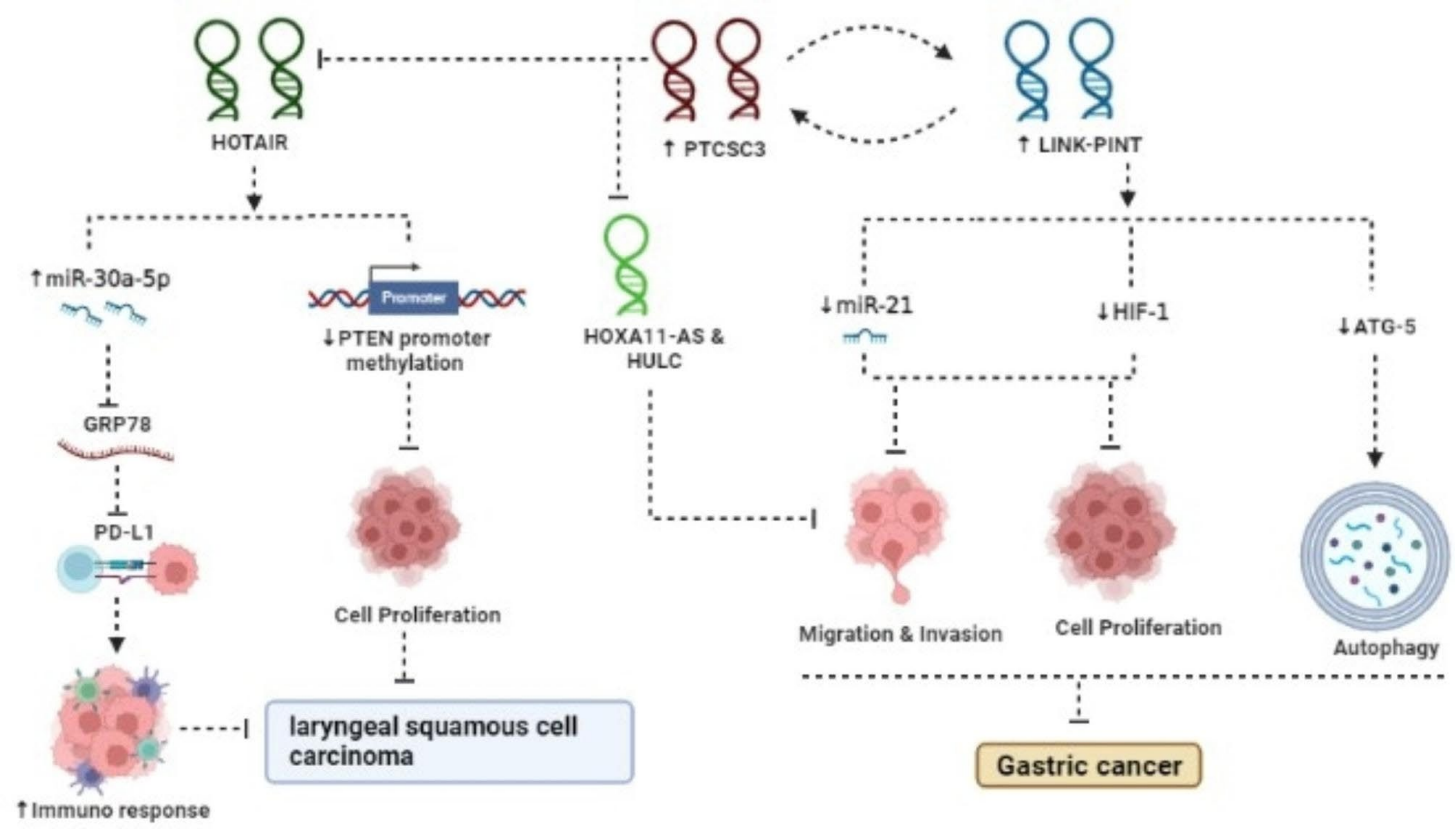
Overexpression of PTCSC3 suppressed progression of cancer cell. Ectopic expression of lncRNA PTCSC3 via changing expression another lncRNAs can effect on different process including: immune response, cell proliferation, invasion, migration, and autophagy

In GC tissues and cell lines, downregulation of PTCSC3 has been observed in several studies [20–23]. The role of lncRNA PTCSC3 in GC is schematically shown in Fig. 2. PTCSC3 functions in a positive feedback regulatory loop with the lncRNA LINC-PINT and low expression levels of these two lncRNAs have been associated with a poor prognosis in patients with GC [21]. The lncRNA LINC-PINT sponges miR-21 in GC cells, leading to the inhibition of proliferation, migration, and invasion of GC cells [24]. Another target of LINC-PINT is HIF‑1α, in which its inhibition following LINC-PINT overexpression causes GC cells to be proliferated slower [25]. LINC-PINT also reduces cisplatin resistance in GC cells by silencing ATG5, a gene that is involved in the process of autophagy activation [26]. It is therefore possible to hypothesize that PTCSC3 through inducing LINC-PINT, inhibits the expression of miR-21, HIF-1, and ATG5, and thereby exerts anti-tumor functions in GC cells. Xu et al. showed that the plasma level of PTCSC3 was lower in GC patients than in healthy individuals, and also patients with distant recurrence had a level of PTCSC3 that was more significantly decreased than those with local or no recurrence. In addition, PTCSC3 was found to be a suppressor of lncRNA HOXA11-AS, with PTCSC3 restoration causing HOXA11-AS to be downregulated, thereby reducing GC cell invasion and migration [22]. LncRNA HOXA11-AS plays an oncogenic role in GC cells by regulating a number of key targets such as miR-124-3p, miR-1297, miR-148a, SRSF1, β-catenin, ITGB3, EZH2, and KLF2 [27–30]. Notably, Zhang et al. found that low PTCSC3 expression levels could not only be used to distinguish early-stage GC patients from healthy individuals, and that patients with low plasma levels had a significantly worse outcome, but that PTCSC3 expression in GC patients increased post-treatment compared to pre-treatment levels, indicating that PTCSC3 has diagnostic and prognostic values in GC patients [23].

### Laryngeal squamous cell carcinoma (LSCC)

Low expression levels of PTCSC3 could be used to distinguish early-stage LSCC patients from healthy individuals. In addition, Xiao et al. reported that overexpression of PTCSC3 in UM-SCC-17 A cells was followed by lower expression levels of both STAT3 and the lncRNA HOTAIR, resulting in decreased LSCC cell proliferation [31]. HOTAIR affects GRP78 stability post-transcriptionally by sponging hsa-miR-30a-5p, acting as a positive regulator of LSCC (Fig. 2). Additionally, HOTAIR increases immune escape by increasing PD-L1 expression via the hsa-miR-30a-5p/GRP78 axis [32]. Besides that, PTEN methylation is one of the epigenetic modifications that is involved in tumor progression; HOTAIR enhances tumor growth in LSCC cells by inducing PTEN methylation [33]. It follows that by inhibiting HOTAIR, PTCSC3 raises the levels of hsa-miR-30a-5p and lowers the levels of GRP78, and PD-L1, as well as reduces PTEN methylation in LSCC cells, which may explain how PTCSC3 inhibits the proliferation of LSCC cells.

### Triple negative breast cancer (TNBC)

Wang et al. indicated that the PTCSC3 level was lower in the tissue and plasma samples of TNBC patients than in control samples. The proliferation of TNBC cells was inhibited by ectopic expression of PTCSC3, but migration and invasion were unaffected. Ectopic expression of PTCSC3 could also downregulate lncRNA H19 in the TNBC cells [34]. Previous studies have shown that lncRNA H19 is strongly associated with breast cancer by regulating important genes and molecular pathways that are involved in the tumorigenesis and progression of the disease [35].

### Cervical carcinoma (CC)

It has been reported that the expression level of PTCSC3 is decreased in the tissue samples and cell lines of CC patients, indicating that it plays a tumor-suppressive role in CC. It has been demonstrated that PTCSC3 directly targets miR-574-5p in CC cells, in which overexpression of PTCSC3 in CC cells causes miR-574-5p to be downregulated. As a result, the proliferation, migration, and invasion of cc cells reduce, while cell cycle arrest enhances. Additionally, PTCSC3 overexpression in CC cells causes E-cadherin to be upregulation while cyclin D1, MMP-9, N-cadherin, and β-catenin are downregulated [36, 37]. Therefore, it’s possible that PTCSC3-induced CC suppression is mediated by miR-574-5p.

### Oral cancer (OC)

Zhang et al. showed that the expression level of PTCSC3 was significantly suppressed in human OC tissues and cell lines. Additionally, PTCSC3’s ectopic expression inhibited the proliferation of OC cells by activating of apoptosis, as evidenced by an increase in Bax and a decrease in Bcl-2. Other target genes that were upregulated after PTCSC3 overexpression included LC3B-I and Beclin-1, both of which are indicators of autophagy activation [38].

### Lung cancer

It has been found that smoking can alter the pattern of lncRNAs expressed in patients with lung squamous cell carcinoma (LUSC) and lung adenocarcinoma (LUAD). A negative correlation between the smoking index and lncRNA PTCSC3 expression has been reported in female smokers with LUAD [39].

### Glioma cancer

**Fig. 3:**
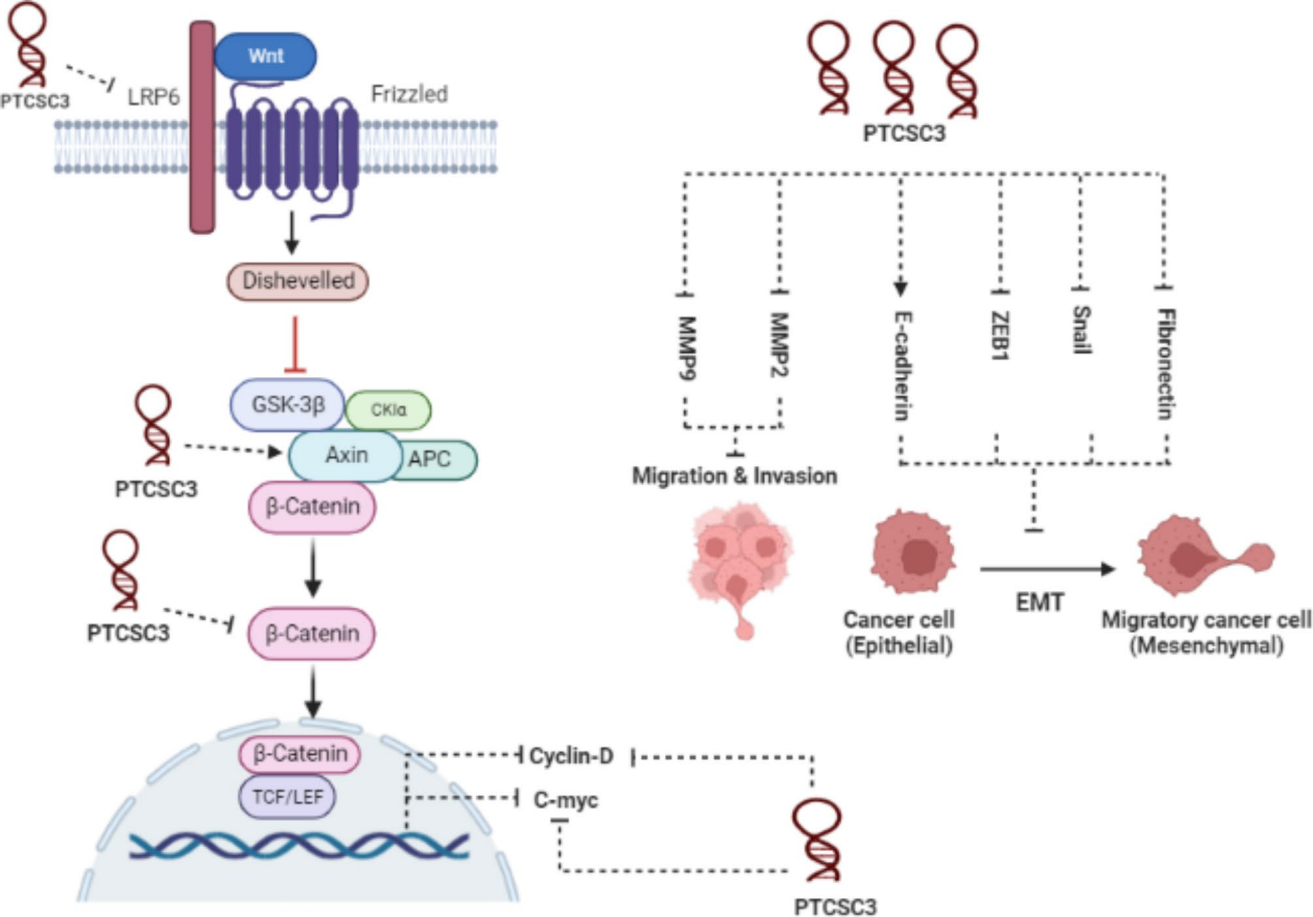
The tumor suppressive role of PTCSC3 in glioma cells. LncRNA PTCSC3 suppresses the activation of Wnt/β-catenin signaling pathway by targeting LRP6 and major players including active β-catenin, and Axin, as well as downstream targets of the pathway including c-myc, and cyclin D1. In addition, PTCSC3 inhibits EMT process in the glioma cells by blocking Snail, ZEB1, and fibronectin, while promoting E-cadherin

The expression level of PTCSC3 in glioma cancer, like in other cancers, shows a decreasing pattern. Xia et al. reported that after overexpression of PTCSC3 in U251 and U87 cells, many characteristics of cancer cells, including proliferation, invasion, migration, and epithelial-mesenchymal transition (EMT) were inhibited (Fig. 3). PTCSC3 exerts these functions via interaction with LRP6, a receptor for the Wnt signaling pathway. Since LRP6 is the target of PTCSC3, after overexpression of PTCSC3 the expression of genes downstream of the pathway including β-catenin, Cyclin D1, and C-myc was suppressed, while the expression of Axin1 was increased [40].

## Non-cancerous disorders

Dysregulation of PTCSC3 has been reported in non-cancerous disorders notably osteoporosis and periodontitis.

### Osteoporosis

Inhibiting the activity of osteoclasts and activating osteoblasts is a common way to treat osteoporosis [41]. PTCSC3 is not only overexpressed in plasma samples of patients with osteoporosis, but it also has a positive correlation with osteoporosis stages. It was found that silencing of PTCSC3 suppressed apoptosis in osteoblast, while its overexpression strongly promoted apoptosis. However, PTCSC3 had no effect on the percentage of apoptosis in osteoclasts [42].

### Periodontitis

Periodontal ligament stem cells (PDLSCs), which are located in the perivascular space of the periodontium, are nowadays considered as a promising tool for the regeneration of periodontal. Liu et al. showed that the expression levels of PTCSC3 and TLR4 were remarkably downregulated and upregulated, respectively, in periodontal PDLSCs isolated from periodontitis- affected teeth in comparison with healthy control. They also revealed suggested a negative correlation between that the expression levels of TLR4 and PTCSC3 were negatively correlated. In PDLSCs, overexpression of PTCSC3 led to the downregulation of TLR4 at both the mRNA and protein levels, as well as, and the inhibition of cell proliferation [2].

## PTCAC3 lncRNA as a diagnostic and prognostic biomarker

PTCSC3 can be used to diagnose and predict the prognosis of certain tumors. In the study of Hong et al., there was a strong correlation between low PTCSC3 and Linc-pint levels and poor survival of patients with GC [25]. In addition, Zhang et al. reported that GC patients who had decreased plasma levels of PTCSC3 exhibited a considerably worse overall survival rate during subsequent follow-up visits. Moreover, it has been proposed that measuring PTCSC3 levels before therapy may help predict how well patients with GC will survive. Therefore, PTCSC3 has diagnostic and prognostic significance in GC patients [23]. In the context of LSCC, Xiao et al. reported that PTCSC3 has the potential to be used as a diagnostic biomarker for the early stage LSCC [31]. Besides that, the expression levels of PTCSC3 in plasma samples had good effectiveness in separation between each stage with healthy control. Therefore, PTCSC3 can be proposed as a potential diagnostic biomarker for patients with osteoporosis [42].

## Association of PTCAC3 with clinicopathological characteristics in cancers

Aberrantly downregulated PTCAC3 was associated with clinicopathological features of cancer (Table 2). Ebrahimi et al. reported that higher levels of PTCSC3 has a significant association with tumors with HER2^−^ status, free-lymph node metastasis (LNM), and early stages of BC [43]. However, another study showed that the comparison of PTCSC3 expression in different stages of cancer do not show any significance in BC [34]. In gastric tumors, PTCSC3 was significantly correlated with stages of tumors, differentiation, tumor diameter, and metastasis [22]. Also, the patients with low plasma levels of PTCSC3 showed a significantly lower overall survival rate compared with patients with the high plasma PTCSC3 level [21, 23]. In comparison between thyroid cancer and peritumoral tissues, the expression levels of PTCSC3 were reduced significantly in all stages but there was no significant difference in PTCSC3 expression between clinical stages (I, II, III, IV), suggesting that PTCSC3 downregulation is an early event in PTC progression [18]. Xiao et al. reported that the plasma level of PTCSC3 was downregulated in the early stage of laryngeal squamous cell carcinoma patients [31]. Taken together, it can be concluded that the expression level of PTCSC3 has a significant association with clinicopathological characteristics in different tumors. It is worth considering that the expression of PTCSC3 is more related to the early stages of tumors.

**Table 2:** The association of the expression of PTCSC3 and clinic-pathological features or prognostic value

Cancer type	PTCSC3 expression	Clinicopathological characteristics	Prognostic/ diagnostic value	Ref
PTC	Down	Reduced in all clinical stages	-	(18)
GC	Down	Associated with poor survival	Prognostic factor	(21)
GC	Down	Associated with clinical stages, Biomarker for the treatment	Prognostic factor	(23)
GC	Down	Associated with tumor diameter, differentiation, metastasis, and recurrence	-	(22)
GC	Down	Associated with clinical stages	-	(20)
TNBC	Down	Associated with clinical stages	-	(43)
LSCC	Down	Associated with clinical early stage	Diagnostic factor	(31)

## Association between PTCAC3 SNPs and risk of human disorders

Some single nucleotide polymorphisms (SNPs) that are associated with the increased risk of human disorders are located near PTCAC3. The most investigated SNP is rs944289 which is located 3.2 kb upstream of PTCSC3 and resided in the binding site for C/EBPα and C/EBPβ transcription factors. The TT genotype of this SNP has conferred the strongest suppression of PTCSC3 expression in PTS, while this genotype was associated with PTCSC3 upregulation in unaffected thyroid tissues. On the other hand, the risk genotype (TT) changes the binding site of C/EBPα and C/EBPβ, which makes them unable to bind to their binding sites and activated PTCSC3 expression [9]. Lee et al. showed that in patients with large-vessel ischemic stroke (a major subtype of ischemic stroke), there are 5 SNPs including rs944289, rs934075, rs2415317, rs1952706, and rs2787417 within PTCSC3 gene. They claimed that PTCSC3 is involved in the pathogenesis of LAA stroke and there is an association between thyroid function and LAA stroke [44]. Another study by Wang et al., showed a significant association between the rs944289 TT genotype and reduced CRC risk in the Han Chinese population [10].

## Conclusion and perspectives

PTCSC3 is now recognized as a hub lncRNA, and many studies have focused on its role in human diseases. PTCSC3 expression has been reported to be decreased in every type of tumor studied to date; it has also been reported to be downregulated in non-cancerous disease, periodontitis, despite being increased in osteoporosis. The downregulation of PTCSC3 in different types of cancers indicates that it plays an important role in the pathogenesis of human cancers. At the same time, PTCSC3 is closely related to the clinical characteristics of tumors. According to a large body of evidence, overexpression of PTCSC3 significantly inhibits tumor progression through a variety of mechanisms, including apoptosis induction, cell cycle arrest promotion, miRNA sponging, suppression of EMT, increasing chemosensitivity of cancer cells, and decreasing cancer cell invasion and migration. Because PTCSC3’s role in various other cancers and other non-cancerous diseases has not been researched, our knowledge of it is currently incomplete. Additional research is required to determine the precise mechanism of PTCSC3 in human disorders. Moreover, PTCSC3 needs to be further studied in the blood of patients with cancer and non-cancer disorders in order to be used in clinical applications. Additionally, it is necessary to assess PTCSC3 expression following chemotherapy treatment in future studies.

## Data Availability

Not applicable.

## Abbreviations

lncRNA: Long non-coding RNA
PTCSC3: Papillary Thyroid Carcinoma Susceptibility Candidate 3
ncRNAs: non-coding RNA
miRNA: microRNA
3'-UTR: Untranslated region
mRNA: Messenger RNAs
RNAPII: RNA polymerase II
SNP: Single Nucleotide Polymorphisms
SCAI: Suppressor of Cancer Cell Invasion
MRTF: Myocardin- Related Transcription Factor
STAT3: Signal Transducer and Activator of Transcription3
PGK1: Phosphoglycerate kinase 1
INO80: INOsitol-requiring mutant 80
MMP-9: Matrix metallopeptidase 9
VEGF: Vascular endothelial growth factor
LINC-PINT: Long intergenic non-protein-coding RNA p53-induced transcript
HIF‑1α: Hypoxia-inducible factor 1-alpha
ATG5: Autophagy related 5
HOXA11-AS: Homeobox A11 antisense
SRSF1: Serine arginine-rich splicing factor 1
ITGB3: Integrin beta 3
EZH2: Enhancer of zeste homolog 2
KLF2: Kruppel like factor 2
HOTAIR: HOX transcript antisense RNA
GRP78: Glucose-regulated protein
PD-L1: Programmed cell death ligand 1
PTEN: Phosphatase and tensin homolog
BAX: Bcl-2-associated x protein
Bcl-2: B-cell lymphoma 2
LRP6: Low-density lipoprotein receptor-related protein 6
